# Evaluation of Node-Inhomogeneity Effects on the Functional Brain Network Properties Using an Anatomy-Constrained Hierarchical Brain Parcellation

**DOI:** 10.1371/journal.pone.0074935

**Published:** 2013-09-18

**Authors:** Bumhee Park, Jeong Hoon Ko, Jong Doo Lee, Hae-Jeong Park

**Affiliations:** 1 Department of Nuclear Medicine and Radiology, and Severance Biomedical Science Institute, Yonsei University College of Medicine, Seoul, Korea; 2 BK21 Project for Medical Science, Yonsei University College of Medicine, Seoul, Korea; 3 Department of Biomedical Engineering, Duke University, Durham, North Carolina, United States of America; Institute of Psychology, Chinese Academy of Sciences, China

## Abstract

To investigate functional brain networks, many graph-theoretical studies have defined nodes in a graph using an anatomical atlas with about a hundred partitions. Although use of anatomical node definition is popular due to its convenience, functional inhomogeneity within each node may lead to bias or systematic errors in the graph analysis. The current study was aimed to show functional inhomogeneity of a node defined by an anatomical atlas and to show its effects on the graph topology. For this purpose, we compared functional connectivity defined using 138 resting state fMRI data among 90 cerebral nodes from the automated anatomical labeling (AAL), which is an anatomical atlas, and among 372 cerebral nodes defined using a functional connectivity-based atlas as a ground truth, which was obtained using anatomy-constrained hierarchical modularity optimization algorithm (AHMO) that we proposed to evaluate the graph properties for anatomically defined nodes. We found that functional inhomogeneity in the anatomical parcellation induced significant biases in estimating both functional connectivity and graph-theoretical network properties. We also found very high linearity in major global network properties and nodal strength at all brain regions between anatomical atlas and functional atlas with reasonable network-forming thresholds for graph construction. However, some nodal properties such as betweenness centrality did not show significant linearity in some regions. The current study suggests that the use of anatomical atlas may be biased due to its inhomogeneity, but may generally be used in most neuroimaging studies when a single atlas is used for analysis.

## Introduction

Graph-theoretical network analysis was initially applied in the analysis of social phenomenon such as the internet [1,2] and social interactions [3,4], but it is now widely used to analyze complex networks of the brain (for review, see 5). This graph theoretical approach to brain as a functional network has been greatly accelerated by the introduction of resting state fMRI (rs-fMRI), which shows clustered slow fluctuations during task-independent resting state [6-8]. In the analysis of functional brain networks using graphs, definition of nodes (i.e., subdivision of meaningfully homogeneous parcels) within the anatomically continuous architecture of the brain is highly important [9,10].

Since the early cytoarchitectonic parcellation by Brodmann [11], various approaches have been undertaken to define subdivisions of the brain. The brain parcellation has become more popular with the advance of structural neuroimaging techniques that has enabled more accessible parcellation of the individual cortex based on the anatomical landmarks of sulcus and gyrus patterns [12-14].

Accordingly, many graph-theoretical analyses of functional brain networks using rs-fMRI have been conducted based on an anatomical atlas that divides the whole brain into 60 to 120 subregions [15-18]. For example, the automated anatomical labeling (AAL) partitions the whole brain into 116 regions including the cerebellum [14], FreeSurfer maps subdivide brain into 78 regions [13,19,20], and the Harvard-Oxford atlas subdivides the whole brain into 68 regions [21]. Although it is relatively simple to consider each anatomical subdivision as a node in the graph analysis, the fundamental assumption of homogeneity within a node is questionable especially when these atlases are applied to functional network analysis.

For instance, the precentral and postcentral gyrus in anatomical atlas can be further segregated into multiple functional subunits specialized for hand, foot, face, lip, tongue, etc. Treating the precentral gyrus as a single functional node may therefore lead to an inaccurate representation of a functional node. Accordingly, inhomogeneity within each region may affect the estimation of the interregional functional connectivity that is generally defined by the temporal correlation between mean rs-fMRI activities of two regions. Incorrect estimation of functional connectivity caused by intra-nodal inhomogeneity may result in erroneous construction of a brain graph.

**Figure 1:**
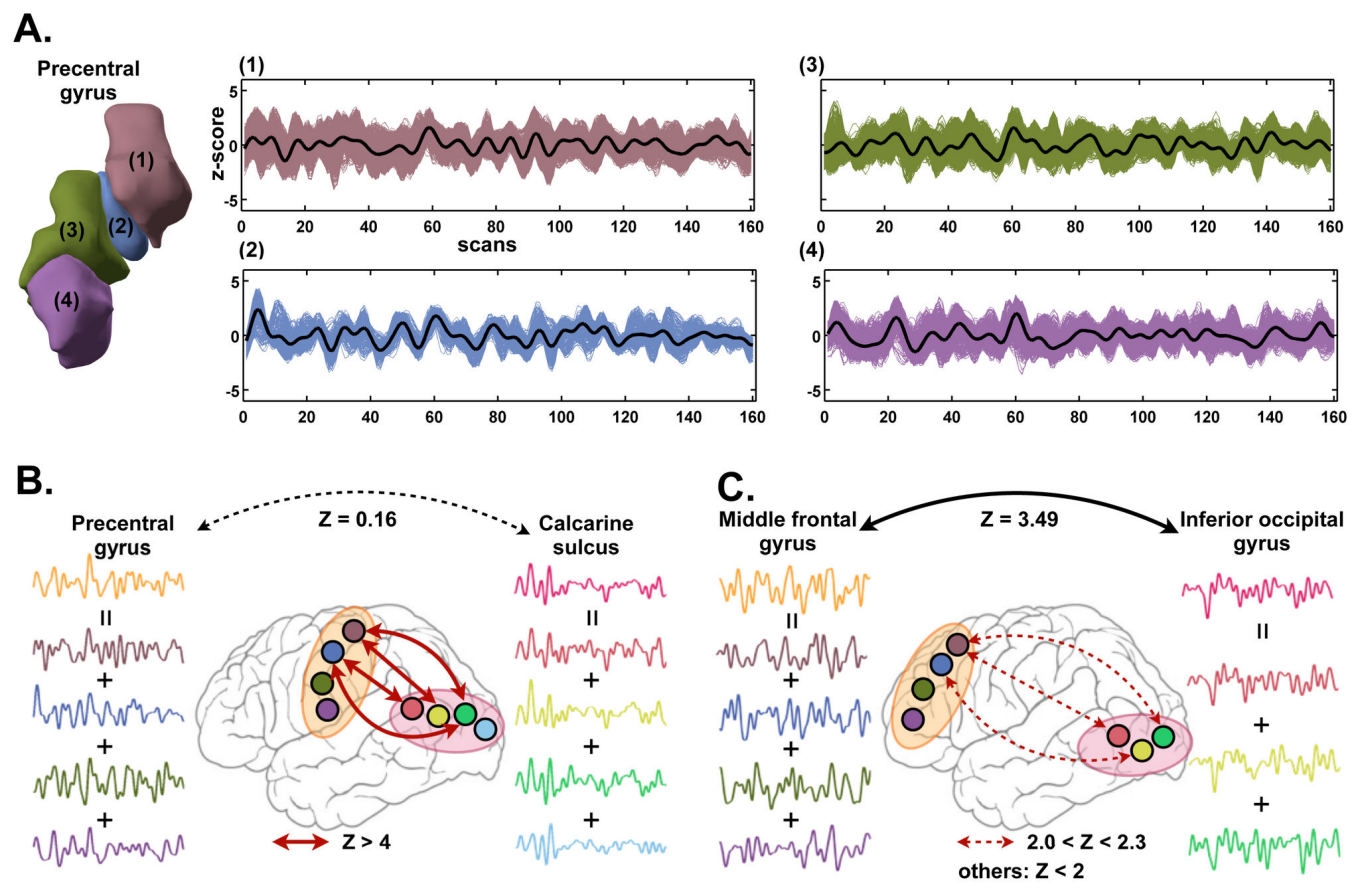
Functional inhomogeneity of the anatomy-based parcellation and its effects on functional connectivity. (A) Four subregions within a precentral gyrus showed different fMRI time activities. (B) Mean time series of the precentral gyrus and calcarine sulcus have low correlation (Z=0.3), but there exist several significant correlations among subregions of them. (C) Mean time series of the precentral gyrus and visual cortex had high correlation (Z=3.49), but in reality there exist only low correlations among smaller subregions when each anatomically defined brain region were broken into smaller subregions by modularity optimization.

Figure 1 presents an example of functional connectivity misled by such inhomogeneity in anatomical atlas. As each anatomical region contains functionally inhomogeneous subregions (e.g., the precentral gyrus in Figure 1A), averaging signals from these subregions to represent the activity of the parent region may lead to a spurious observation. For example, we may not observe a significant connectivity between the precentral and calcarine sulcus (Figure 1B) despite the existence of significant connectivity between subregions of two regions. The opposite case, in which the subregions may not have significant correlation but their parent nodes do, may also hold true for some regions (Figure 1C). In line with this example, how functionally inhomogeneous nodes affect the graph topological properties is the motivation of the current study.

The current study was aimed to show functional inhomogeneity of a node defined by anatomical atlas and to show the effects of functional inhomogeneity on the graph topology. Among several anatomical atlases, we evaluated the AAL since it is currently widely used for brain network analysis [15-18,22]. We assumed that results would not be significantly affected by the choice of anatomical atlas for the purpose of our study. To compare anatomically defined nodes with functionally homogenous nodes, we used a functional atlas derived by proposing an anatomy-constrained hierarchical modularity optimization algorithm (AHMO) to rs-fMRI. Using this algorithm, we subdivided each ROI in the AAL map into several functionally homogeneous subunits and evaluated the effects of inhomogeneity on the functional connectivity and functional network properties by comparing the AAL and the AHMO maps, assuming the functional map to be a gold standard.

In summary, we evaluated the effect of node definition on the inter-regional functional connectivity, and global/local graph properties. We also evaluated the reliability of using an anatomical atlas (AAL map in this study) in systematic applications (e.g., group comparison or correlation analysis using the same anatomical atlas) by calculating the linearity between global graph properties derived from anatomical and functional atlases. We conducted a group comparison of global and local graphical properties between male and female to evaluate the interaction effect between group and atlas. Additionally, we evaluated the effects of the parcellation methods, the definition of the time series for brain regions, and thresholds in constructing weighted graphs.

## Materials and Methods

### Subjects

138 healthy right-handed volunteers (mean age=24.2±3.3; 74 males and 64 females) participated in this study. No participants had a history of neurological illness or psychiatric disorders. Handedness was assessed with a Korean version of the Annett handedness questionnaire [23]. All participants gave written informed consent for participation according to the Declaration of Helsinki (BMJ 1991; 302: 1194) and this study was approved by the Severance Institutional Review Board (IRB).

### Image acquisition

All participants underwent fMRI scanning with a 3.0 Tesla MRI scanner (Philips Achieva, Philips Medical System, Best, The Netherlands) to obtain T2* weighted single shot echo planar imaging (EPI) axial scans with the following parameters: voxel size, 2.75 x 2.75 x 4.5 mm3; slice number, 29 (interleaved); matrix, 80×80; slice thickness, 4.8 mm; repetition time (TR), 2000 ms; echo time (TE), 30 ms; and field of view, 209×220 mm^2^. To facilitate later spatial normalization, we also obtained a high resolution T_1_-weighted MRI volume data set for each subject using a three-dimensional T1-TFE sequence configured with the following acquisition parameters: voxel size; 0.859 x 0.859 x 1.2 mm3, TR; 9.6 ms, TE; 4.6 ms. Foam pads were used to reduce head motion during EPI data acquisition.

For rs-fMRI data, we acquired functional scans while participants lay resting with eyes closed without focusing any specific thoughts and without sleep, which was evaluated by a questionnaire after scanning. It consisted of 165 volumes per participant, which took approximately 5.5 minutes.

### Image preprocessing

We preprocessed for rs-fMRI using statistical parametric mapping (SPM8, Wellcome Department of Cognitive Neurology, London, UK) [24]. This process included correction for acquisition time delay between different slices, and correction for head motion by realigning all consecutive volumes to the first image of the session. The realigned images were co-registered to T1-weighted images, which were then used to spatially normalize functional data into a template space using nonlinear transformation. Finally, we spatially smoothed all normalized images using a 6 mm full-width half-maximum Gaussian kernel.

For rs-fMRI analysis, we discarded the first 5 scans for stability issues and used the 160 EPI data for analysis. We used the canonical signal processing procedures for calculating the resting state functional connectivity as in a previous study [25]. More specifically, fMRI data were band-pass filtered (0.009–0.08 Hz) and effects of six rigid motion and global signal changes in white matter, cerebrospinal fluid, and whole brain were regressed out.

### Anatomy-constrained hierarchical modularity optimization (AHMO)

**Figure 2:**
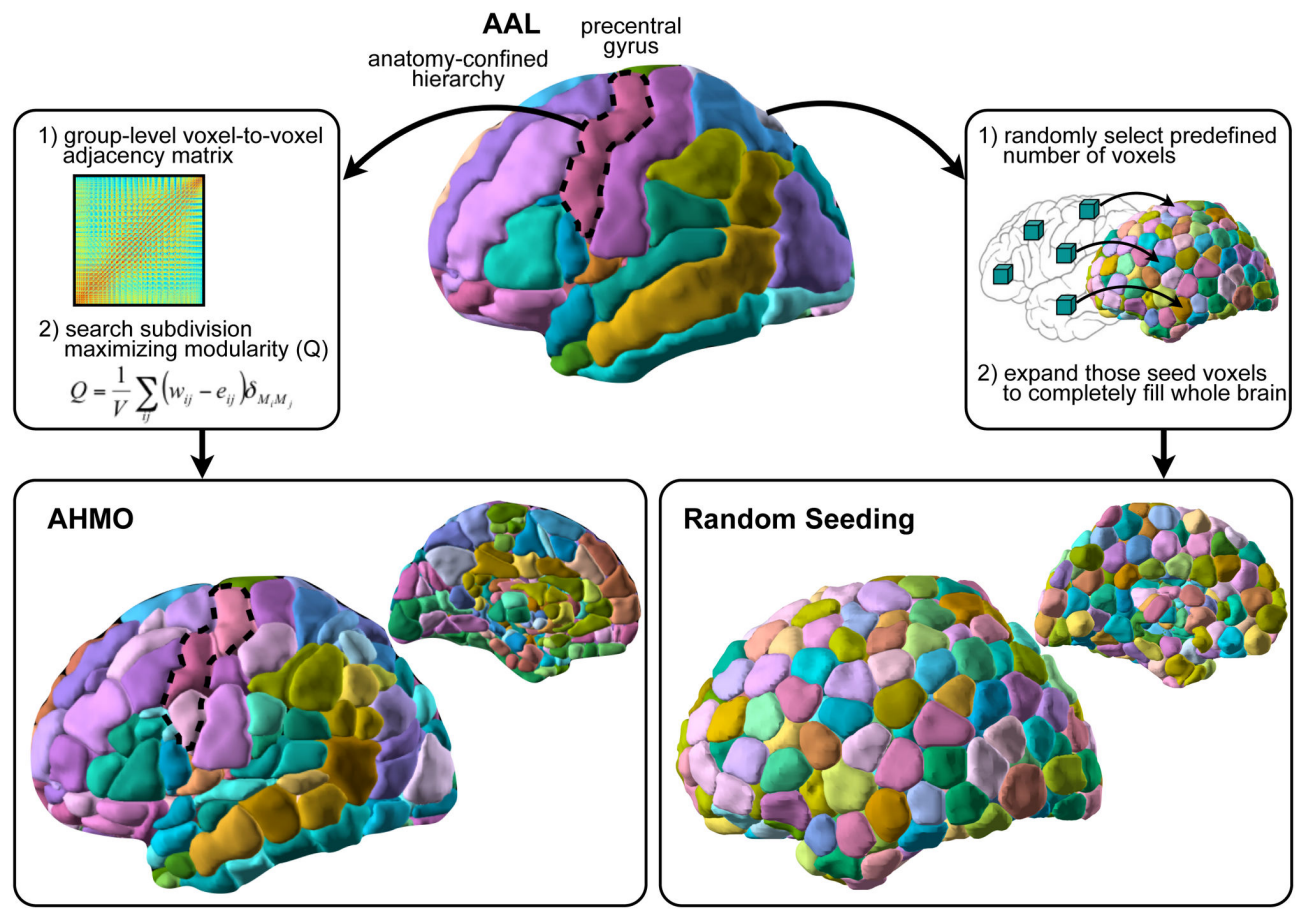
Description of AHMO and random seeding method. AHMO applies voxel-level modularity optimization for each individual region in an anatomical atlas, while random seeding method assigns pre-defined number of seeds to random location within a region and grows the seeds with maximally uniform size until the whole region is populated with the seed clusters (Zalesky et al., 2010). Colors for visualization were selected by algorithm developed by the BrainCOLOR project (http://www.braincolor.org/). Note that AHMO maintains the initial AAL ROI contiguity.

To generate functionally homogeneous atlas from the given anatomical atlas, we proposed a hierarchical parcellation algorithm based on modularity optimization. Modularity (*Q*) is defined as (total connection weights bounded by subdivisions) – (chance-expected total connection weights), and modularity optimization searches for region-partitioning that maximizes *Q* [26]. The basic idea of the proposed AHMO is to apply modularity optimization to each region of interest (ROI), i.e., a subregion in the anatomical atlas, to partition the anatomical ROI into functionally homogeneous subregions (Figure 2). This anatomically contained partitioning method is useful for comparing graph properties with those of AAL method. The method for AHMO can be summarized as below:

1 For a given anatomical ROI, voxel-to-voxel correlation coefficients among times series of voxels within the ROI were calculated to construct an adjacency matrix (correlation matrix) for the ROI. In this case, all voxels in the ROI serve as nodes in the graph.
2 Group-averaged adjacency matrix was generated after Fisher’s r-to-z transformation of individual adjacency matrix in a group (n=138, the number of subjects). We multiplied all z-values by $\sqrt{Ns-3}$ to standardize each z-value, i.e., *N*(0,1), where *Ns* is the temporal sample size 160 as the number of scans.
3 Modularity optimization was applied to the group-averaged adjacency matrix to cluster the voxels within the ROI into several subregions.

This step was repeated for 90 cortical ROIs in the AAL atlas. This concept can be mathematically represented as follows:

$$Q_{k}=\frac{1}{V_{k}}\sum_{ij}(w_{ij}^{k}-e_{ij}^{k})\delta_{C_{i}^{k}C_{i}^{k}}$$

$$e_{ij}^{k}=\frac{s_{i}^{k}s_{j}^{k}}{V_{k}},s_{i}^{k}=\sum_{j}w_{ij}^{k},V_{k}=\sum_{ij}w_{ij}^{k}$$

where $e_{ij}^{k}$ is the chance-expected total weight, $w_{ij}^{k}$is the connection weight between voxel *i* and *j* in the *k*-th ROI in the AAL, *V*_*k*_ is the total sum of weights rescaling *Q*_*k*_ to [0,1], and $\delta_{C_{i}^{k}C_{i}^{k}}$ is the indicator whose value is one if voxel *i* and voxel *j* in the ROI *k* are in the same cluster (*C_i_ = C_j_*) and zero otherwise. The number of clusters in the ROI *k* is determined by selecting a subdivision maximizing *Q* among the set of all possible subdivisions.

After this process, we labeled spatially unconnected subclusters within a cluster as new clusters and then combined small-sized clusters (<50 contiguous voxels) to the closest clusters (center-to-center).

### Evaluation of network properties defined using AAL and AHMO

We calculated functional connectivity and local (or nodal) and global properties in both AAL and AHMO to investigate the effects of functional inhomogeneity within an ROI on these indices.

### 1) Functional Connectivity

We defined functional connectivity between ROIs using correlation coefficient between the mean time series of two ROIs. In order to compare functional connectivity in the AAL and AHMO at the level of the AAL ROIs, we further defined a mean sub-regional functional connectivity between sets (i.e., AAL ROIs) of AHMO subregions as follows:

$$r_{kl}^{AHMO}=\frac{1}{n_{k}n_{l}}\sum_{i\in C_{k}}\sum_{j\in C_{l}}r(x_{i}^{k},x_{j}^{l})$$

where C_k_ and C_l_ are sets of AHMO subregion indices corresponding to the *k-*th and *l-*th AAL ROIs, *n*_*k*_ and *n*_*l*_ are the size of those sets (For AAL, *n*_*k*_ =1 and *n*_*l*_ =1), and *x*_*i*_^(k)^ and *x*_*j*_^(l)^ are mean time series of the *i-*th subregion within the *k-*th AAL ROI and the *j*-th subregion within the *l-*th AAL ROI, respectively.

After Fisher’s r-to-z transformation of individual adjacency matrices, we compared all functional connectivity pairs between AHMO and AAL using paired t-test.

### 2) Local graph properties

We calculated two nodal topological measures (nodal strength and betweenness centrality in weighted graph) [27] using adjacency matrices derived from the AAL (90 x 90) and AHMO (372 x 372), after thresholding those matrices at a network-forming threshold of *Z*=3.

Nodal strength of the *i*-th node was defined as the sum of all connection weights between the node and the other nodes:

$$s_{i}=\sum_{j\in N}z_{ij}$$

where *z*_*ij*_ is the z-score of correlation coefficient between the *i*-th and *j*-th nodes in the AAL or AHMO and **N** is the set of all nodes in the graph. Betweenness centrality of the *i*-th node was defined as the fraction of all shortest paths in the network that pass through the node:

$$b_{i}=\frac{1}{\left(n-1)(n-2\right)}\sum_{\begin{array}{c} \;h,j\in N\\ h\neq j,h\neq i,j\neq i \end{array}}\frac{\rho_{hj}(i)}{\rho_{hj}}$$

where *ρ*_*hj*_ is the number of shortest paths between nodes *h* and *j*, *ρ*_*hj*_(*i*) is the number of shortest paths between nodes *h* and *j* that pass through node *i*, **N** is the set of all nodes in the graph, and *n* is the number of nodes.

We averaged nodal strength and betweenness centrality across AHMO subdivisions for each AAL ROI to make these AHMO measures comparable to the AAL measures. In order to further account for the difference in graph sizes (i.e., number of nodes), we normalized these averaged measures across AAL ROI regions to scale the effect of graph sizes:

$$\begin{array}{c} u_{}^{\left(k\right)}=\frac{1}{n_{k}}\sum_{i\in C_{k}}m_{i}^{\left(k\right)}\\ z_{}^{\left(k\right)}=\left(u_{}^{\left(k\right)}-E\left(\left\{u_{}^{\left(1\right)},u_{}^{\left(2\right)},\cdots ,u_{}^{\left(K\right)}\right\}\right)\right)/\sigma \left(\left\{u_{}^{\left(1\right)},u_{}^{\left(2\right)},\cdots ,u_{}^{\left(K\right)}\right\}\right),\;k=1,...,K \end{array}$$

where C_k_ is the set of the *k-*th AHMO subregion indices corresponding to the *k-*th AAL ROIs, *n*_*k*_ is the size of the set (for AAL, *n*_*k*_ =1), *m*_*i*_^(k)^ can either be nodal strength (*s_i_*) or betweenness centrality (*b_i_*) of the *i*-th AHMO subregions within the AAL region *k*, *E* and σ indicate the mean and standard deviation among averaged nodal measures for all AHMO subregions, and *K* is the total number of AAL regions (*K*=90). We also normalized nodal strength and betweenness centrality of AAL across regions.

We compared the rescaled nodal measures in the AHMO with those in the AAL using paired t-test.

### 3) Global graph properties

We calculated global and local efficiencies [27] using adjacency matrices from AAL (90 x 90) and AHMO (372 x372). Global efficiency is the average of inverse of characteristic path length and can be regarded as a measure of communication efficiency in the brain graph:

$$E_{glob}(G)=\frac{1}{n(n-1)}\sum_{i\in N}\sum_{\begin{array}{c} j\in N\\ j\neq i \end{array}}\frac{1}{d_{ij}}$$

where N is the set of all nodes in the graph *G*, *n* is the number of nodes, and *d*_*ij*_ is the shortest path length between nodes *i* and *j*. Local efficiency is a global efficiency calculated on the neighborhood subgraph of each node:

$$E_{loc}(G)=\frac{1}{2}\sum_{i\in N}\frac{1}{k_{i}(k_{i}-1)}\sum_{\begin{array}{c} j,h\in N\\ j\neq i \end{array}}{\left(w_{ij}w_{ij}d_{jh}{}^{-1}(n_{i})\right)}^{1/3}$$

where N is the set of all nodes in the graph *G*, *k*_*i*_ is the degree of node *i*, *w*_*ij*_ is the connection weight between nodes *i* and *j*, and *d*_*jh*_(*n_i_*) is the shortest path length between nodes *j* and *h* that are neighbors of *i*. We compared each global measurement in the AHMO with that in the AAL using paired t-test.

### Linearity and group x atlas interaction effect between AAL and AHMO

We calculated the linearity between local and global graph properties by using anatomical (AAL) and functional (AHMO) atlases. The linearity between two paired maps was quantified by Pearson’s correlation coefficient. To investigate the effect of inhomogeneity in the anatomical atlas (based on the assumption that AHMO serves as a ground truth) on the group-comparison, we evaluated the sex-atlas interaction using full factorial ANOVA design considering one within-subject factor (atlas), one between-subject factor (sex), and their interaction factor.

### Effects of network sizes, time series and network-constructing thresholds selection

As the size of AAL (n=90) was smaller than the size of AHMO (n=372), the differences in graph properties between two methods may be attributable to the network size rather than to the inhomogeneity. To evaluate the network size versus inhomogeneity effects on graph analysis according to parcellation methods, we generated multiple anatomical atlases with different sizes using a random seeding algorithm, which was proposed by Zalesky et al. [28] (Figure 2). Briefly, we assigned pre-defined numbers of seeds randomly in the gray matter of the whole brain. Seeds grow with a uniform speed until the whole gray matter region is populated with the seed clusters. The number of random seeds was set to 137, 184, 231, 278, 325, or 372 nodes, which were selected to span the range of our experimental atlases (90 nodes for AAL and 372 nodes for AHMO) in increments of 47 nodes. Note that the parcellation method using random seeding is not based on functional homogeneity but based on anatomical Euclidean distance and depends on initial locations of seeds. Hence, the locations of nodes will vary across different trials, and we used this method only for evaluating global properties of the graph.

To compare graph properties of the random seeding map and AHMO in terms of within-subcluster homogeneity, we defined a homogeneity index as the mean of the regional average of within-cluster connectivities (i.e., mean of all voxel-to-voxel connectivities within a subcluster) as follows [29]:

$$H=\frac{1}{K}\sum_{k=1}^{K}\left[\frac{1}{M_{k}(M_{k}-1)}\sum_{i,j\in V_{k},\;i\neq j}z_{ij}^{\left(k\right)}\right]$$

where *z*_*ij*_^(k)^ is the z-score of correlation coefficient between the *i*-th and *j*-th voxels within the subregion *k*, **V_k_** is the set of voxel indices within subcluster *k* in the AHMO or random seeding map, *M*_*k*_ is the size of *V*_*k*_, and *K* is the total number of subclusters (*K*=372 for AHMO and 137, 231 and 372 for random seeding methods). Higher *H* represents more homogeneity. We compared this index between two parcellation methods using paired t-test and correlation analysis against their global and local efficiencies.

We evaluated the effect of time series definition for a ROI in constructing a brain graph. We compared the mean time series across voxels within an ROI to the first eigenvariate time series of the ROI used in SPM voxel-of-interest selection [30]. The first eigenvariate represents the weighted mean time series of the ROI with maximum possible variance.

Most of evaluations were based on a graph thresholded by a network-forming threshold of Z=3 in this study. However, we further evaluated the effect of the network-forming threshold on the linearity between AAL and AHMO on the graph properties, by comparing linearity produced by four different network-forming thresholds at Z=0, 1, 2, and 3.

## Results

Functional parcellation using AHMO and random seeding method resulted in total of 372 partitions in the cerebrum (Figure 2). In AHMO, functional partitions were bounded by the larger anatomical partitions (i.e., AAL). Thus, AHMO does not change contiguity of the initial AAL ROIs.

### Functional connectivity

**Figure 3:**
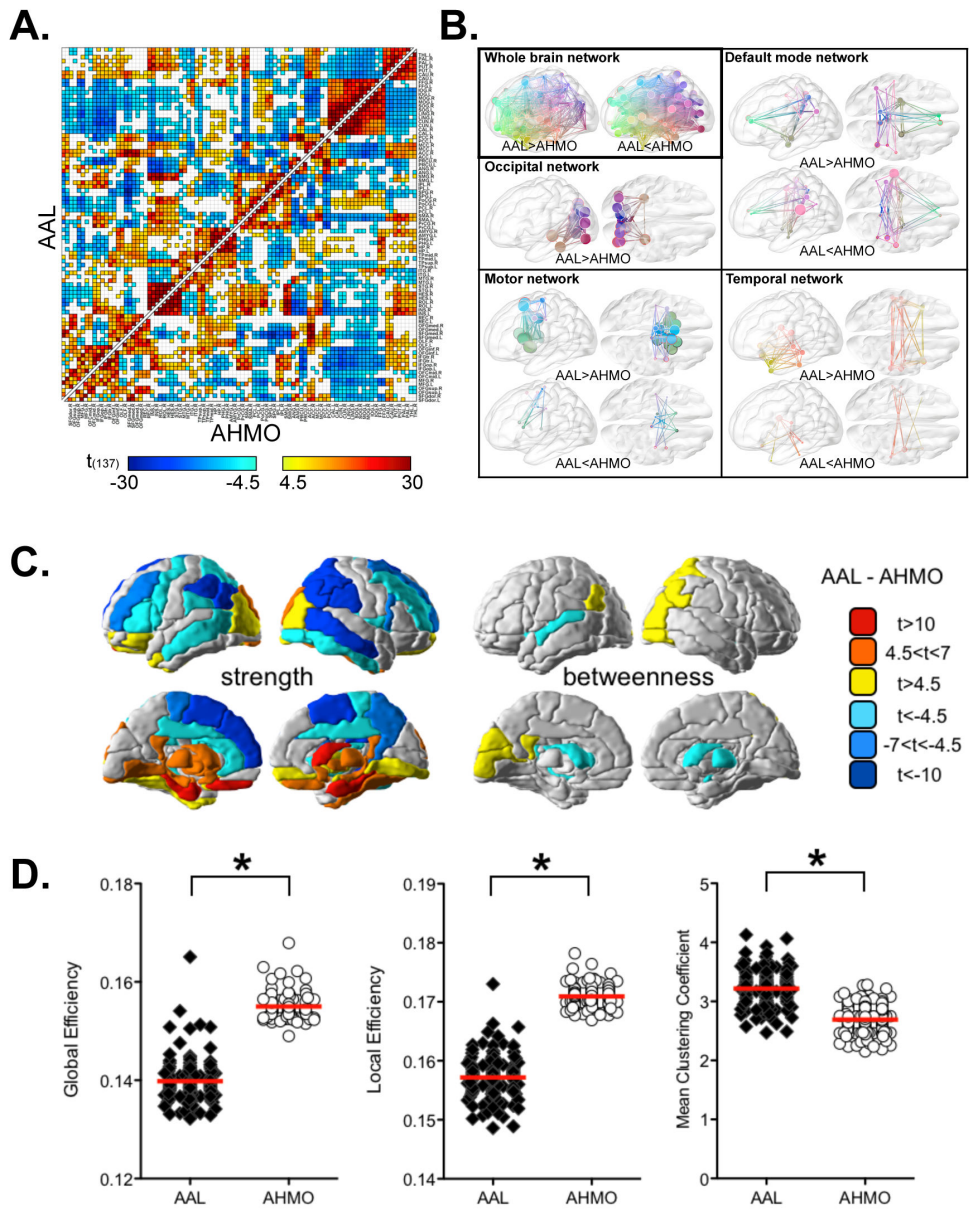
Biased estimation of functional connectivity and graph theoretical properties in the AAL. (A) Elements in upper- and lower- triangular matrix represent functional connectivity maps (adjacency maps) using the AAL and AHMO (regionally averaged corresponding to the AAL map). (B) Functional connectivity difference between the AAL and AHMO. (C) Statistical difference in nodal strength and betweenness centrality between the AAL-based network and AHMO-based network. (D) Global and local efficiency are lower in the AAL than in the AHMO, while mean clustering coefficient is higher in the AAL than in the AHMO. Significance was determined at p<0.05 (Bonferroni corrected for all comparisons).

Functional connectivity difference between the AAL and AHMO is presented in Figure 3 (p<0.05, Bonferroni corrected for all comparisons). Figure 3B shows representative connections such as connections within the default mode network, motor network (including the basal ganglia-thalamus), occipital network, and temporal network.

In the AAL-based network, functional connectivities within the default mode network were estimated to be higher among the posterior cingulate cortex, anterior cingulate cortex, precuneus, angular gyrus, and hippocampus than those in the AHMO-based network. We found higher estimation of functional connectivity in the motor network of the AAL, which consisted of the postcentral gyrus, precentral gyrus, paracentral lobule, and supplementary motor area. These motor regions in the AHMO had lower functional connectivities with the posterior cingulate cortex compared to their AAL counterparts. Most of connectivities related with the basal ganglia and thalamus, the occipital lobe and the temporal lobe were higher in the AAL-based motor network than in AHMO-based network. Lower values of the functional connectivity were found in a circuit related with the supramarginal gyrus and inferior parietal lobule. Results of detailed connections were listed in Figure 3B.

### Local and global graph properties

**Figure 4:**
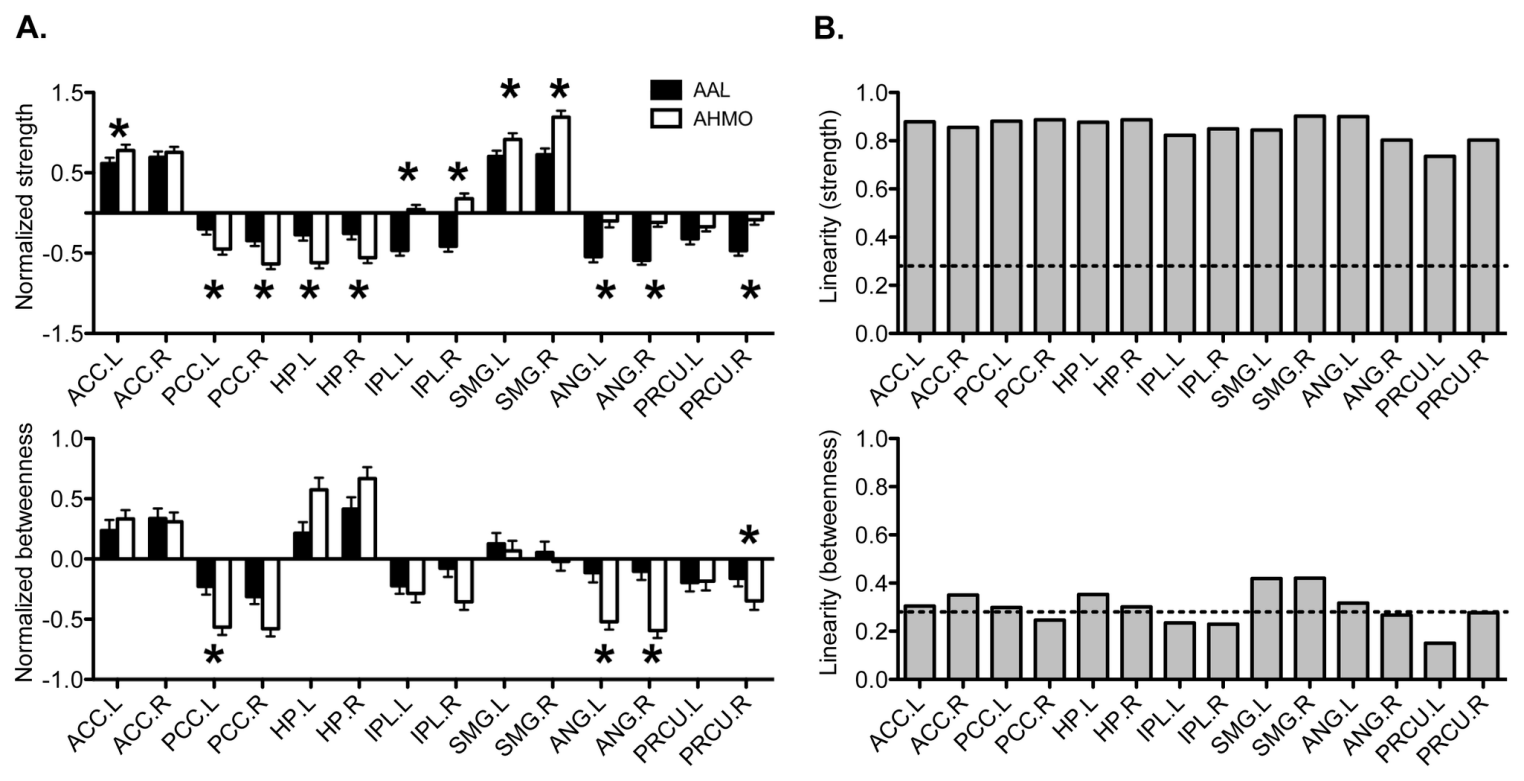
Nodal properties of regions within regions associated with the default mode network. (A) Comparisons of normalized strength and betweenness centrality between AAL and AHMO. Star (*) represents significant difference (P<0.05, Bonferroni corrected for all comparisons). (B) Linearity of strength and betweenness centrality between AAL and AHMO. Dotted line (---) represents significant correlation (R=0.28; P<0.05, Bonferroni corrected for all comparisons). ACC.L: the left anterior cingulate cortex, ACC.R: the right anterior cingulate cortex, PCC.L: the left posterior cingulate cortex, PCC.R: the right posterior cingulate cortex, HP.L: the left hippocampus, HP.R: the right hippocampus, IPL.L: the left inferior parietal lobule, IPL.R: the right inferior parietal lobule, SMG.L: the left supramarginal gyrus, SMG.R: the right supramarginal gyrus, ANG.L: the left angular gyrus, ANG.R: the right angular gyrus, PRCU.L: the left precuneus, PRCU.R: the right precuneus.

All evaluation results of local network properties were presented in Figure 3C and Table 1 (p<0.05, Bonferroni corrected for all comparisons). Figure 4A summarizes evaluation results of network properties in regions within the default mode network. Among the regions, the bilateral posterior cingulate cortex and bilateral hippocampus showed higher strengths, while the left anterior cingulate cortex, bilateral inferior parietal lobule, bilateral supramarginal gyrus, bilateral angular gyrus, and right precuneus showed lower strengths in the AAL-based network (Figure 4A). Betweenness centrality of the left posterior cingulate and bilateral angular gyrus were estimated to be higher in the AAL-based network, while it was underestimated for the left hippocampus compared to those in the AHMO-based network (Figure 4A).

**Table 1:** Statistical differences in local properties.

**Strength**			
**Related network**	**Regions**	**t(137**)	**p-value**
Defaut mode network	Left posterior cinculate cortex	7.4	1.3E-11
	Right posterior cinculate cortex	9.1	1.2E-15
	Left hippocampus	9.8	1.7E-17
	Right hippocampus	8.9	3.5E-15
	Left anterior cingulate cortex	-4.5	1.5E-05
	Left inferior Parietal lobule	-13.8	9.7E-28
	Right inferior Parietal lobule	-16.0	4.4E-33
	Left supramarginal gyrus	-5.0	1.5E-06
	Right supramarginal gyrus	-13.2	2.7E-26
	Left angular gyrus	-12.9	2.1E-25
	Right angular gyrus	-13.9	5.3E-28
	Right precuneus	-9.8	2.2E-17
Occipital network	Left lingual gyrus	9.9	9.7E-18
	Right lingual gyrus	6.9	1.5E-10
	Left superior occipital gyrus	9.4	1.8E-16
	Right superior occipital gyrus	7.3	2.6E-11
	Left middle occipital gyrus	6.8	2.4E-10
	Right middle occipital gyrus	4.5	1.3E-05
	Left fusiform gyrus	6.2	6.1E-09
	Right fusiform gyrus	9.2	4.0E-16
	Right inferior occipital gyrus	-5.1	9.2E-07
Motor and Basal ganglia/Thalamus networks	Left caudate	7.1	6.5E-11
	Right caudate	10.6	1.3E-19
	Left putamen	8.1	2.1E-13
	Right putamen	6.4	2.9E-09
	Left palladium	9.9	8.3E-18
	Right palladium	9.9	7.9E-18
	Left thalamus	7.1	7.2E-11
	Right thalamus	8.5	2.5E-14
	Left paracentral lobule	-8.5	3.0E-14
	Right paracentral lobule	-4.6	9.4E-06
	Left precentral gyrus	-5.9	2.2E-08
	Left supplementary motor area	-12.9	1.9E-25
	Right supplementary motor area	-10.3	7.2E-19
	Left middle cingulate cortex	-5.5	1.8E-07
	Right middle cingulate cortex	-5.4	3.4E-07
Frontal network	Left superior orbitofrontal gyrus	6.8	2.6E-10
	Right superior orbitofrontal gyrus	3.8	2.1E-04
	Left inferior orbitofrontal gyrus	5.1	9.6E-07
	Left olfactory	8.2	1.4E-13
	Right olfactory	9.7	2.3E-17
	Left medial orbitofrontal gyrus	6.6	6.6E-10
	Left rectus	12.4	3.1E-24
	Right rectus	9.1	1.2E-15
	Right dorsal superior frontal gyrus	-8.1	3.5E-13
	Left middle frontal gyrus	-8.2	1.8E-13
	Right middle frontal gyrus	-6.3	4.3E-09
	Opercular part of left inferior frontal gyrus	-3.8	1.8E-04
	Opercular part of right inferior frontal gyrus	-7.2	3.6E-11
	Triangular part of left inferior frontal gyrus	-5.2	7.8E-07
	Left medial superior frontal gyrus	-10.2	1.3E-18
Temporal network	Left middle temporal pole	4.1	7.2E-05
	Right rolandic cortex	-4.9	2.6E-06
	Left middle temporal gyrus	-6.8	3.6E-10
	Right middle temporal gyrus	-12.2	1.5E-23
	Right inferior temporal gyrus	-4.0	1.1E-04
Others	Left amygdala	7.7	2.5E-12
	Right amygdala	6.7	5.4E-10
	Left insula	-4.5	1.7E-05
	Left superior parietal gyrus	-5.8	3.7E-08
	Right superior parietal gyrus	-11.7	2.6E-22
**Betweenness Centrality**			
**Related network**	**Regions**	**t(137**)	**p-value**
Default mode network	Left posterior cinculate cortex	4.3	3.4E-05
	Left angular gyrus	4.7	5.2E-06
	Right angular gyrus	6.1	9.8E-09
Occipital network	Left calcarine sulcus	4.5	1.6E-05
	Left cuneus gyus	4.7	5.2E-06
	Right middle occipital gyrus	3.9	1.4E-04
	Right inferior occipital gyrus	4.7	7.3E-06
Basal Ganglia/Thalamus network	Left caudate	-3.9	1.8E-04
	Right caudate	-4.5	1.5E-05
	Right thalamus	-4.8	3.6E-06
Others	Right amygdala	-4.1	8.1E-05
	Left insula	-3.6	4.9E-04
	Right superior parietal gyrus	5.8	3.7E-08
	Left superior temporal gyrus	-3.8	2.2E-04

Statistical difference (AAL - AHMO) in nodal strength and betweenness centrality between the AAL-based network and AHMO-based network. Significance was determined at p<0.05 (Bonferroni corrected for all comparisons). Positive t-value: AAL>AHMO, negative t-value: AAL<AHMO.

The AAL-based network had lower global (p=3.2e-109) and local efficiencies (p=7.8e-99) and higher mean clustering coefficient (p=8.2e-82) compared to the AHMO-based network (Figure 3D).

### Linear relationship between AAL and AHMO

**Figure 5:**
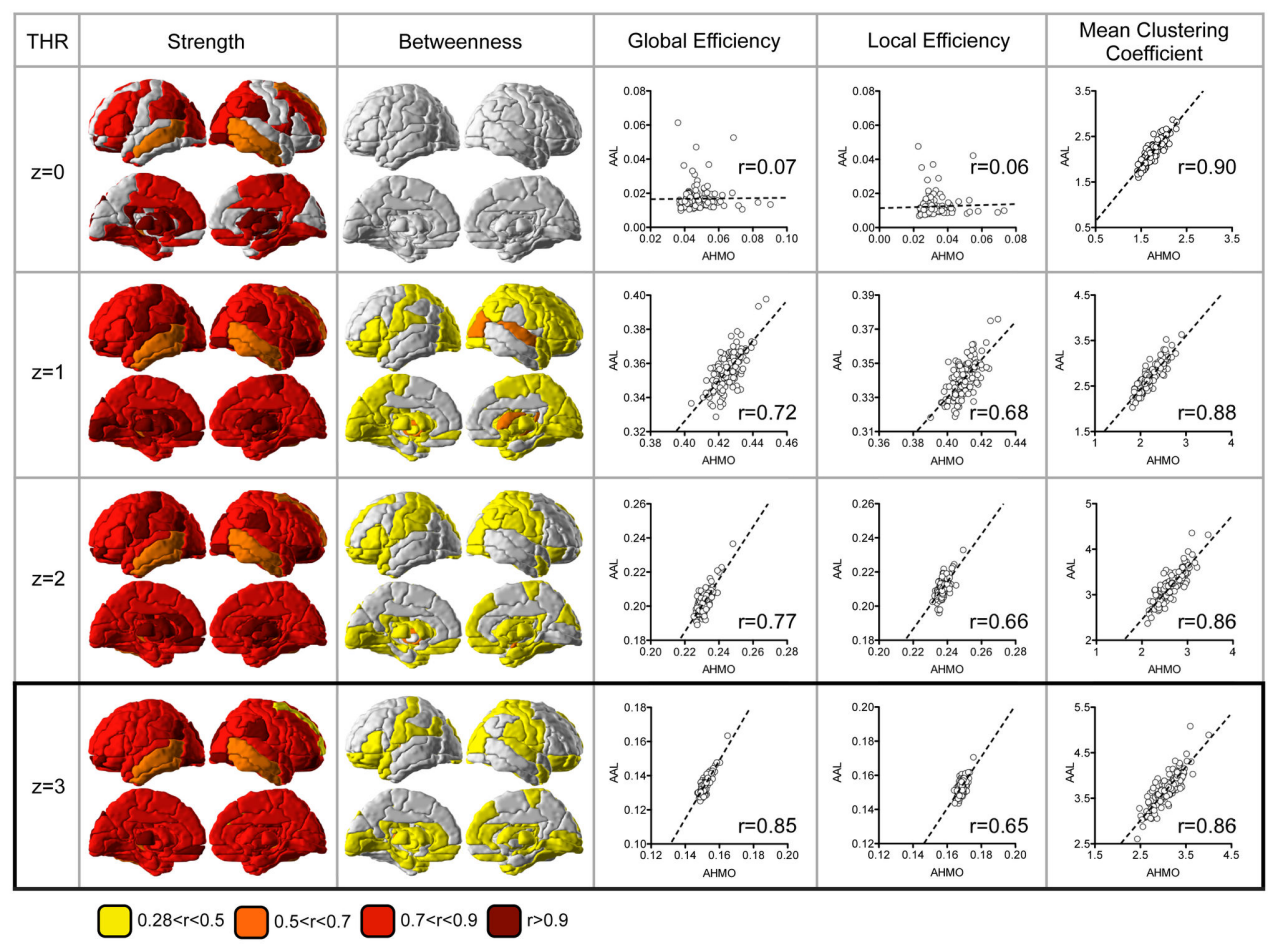
Linearity between AAL and AHMO according to network-forming thresholds. At different network-forming thresholds of Z=0, 1, 2, and 3, the AAL-based and AHMO-based approaches showed significant linearity in nodal strengths in all regions except for betweenness centrality in some regions. Global and local properties showed high linearity between the AAL and AHMO methods regardless of different thresholds of Z=0, 1, 2, and 3.

Figure 5 shows the linearity between nodal strengths and betweenness centralities in the AAL-based network and AHMO-based network across the subjects (r=0.28; p<0.05, Bonferroni corrected for all comparisons). Nodal strengths between the two maps were linear in all regions, while betweenness centralities were linear mainly in some regions within the default mode network, sensory-motor regions, occipital regions, temporal regions, and subcortical regions. Especially, we found that regions within the default mode network (the bilateral anterior cingulate cortex, left posterior cingulate cortex, bilateral hippocampus, bilateral supramarginal gyrus, and left angular gyrus) had highly linear graph theoretical properties between AAL-based network and AHMO-based network (r>0.85) (Figure 4B).

Global network properties in the AAL-based network were plotted against those of the AHMO-based network in order to quantify the linearity with a network-forming threshold of Z=3 (Figure 5). Linearity was relatively low in local efficiency (r=0.65, p=5e-18), but was high in global efficiency (r=0.85, p=2e-39) and in mean clustering coefficient (r=0.86, p=3e-41).

### Interaction between sex and atlas factors

**Figure 6:**
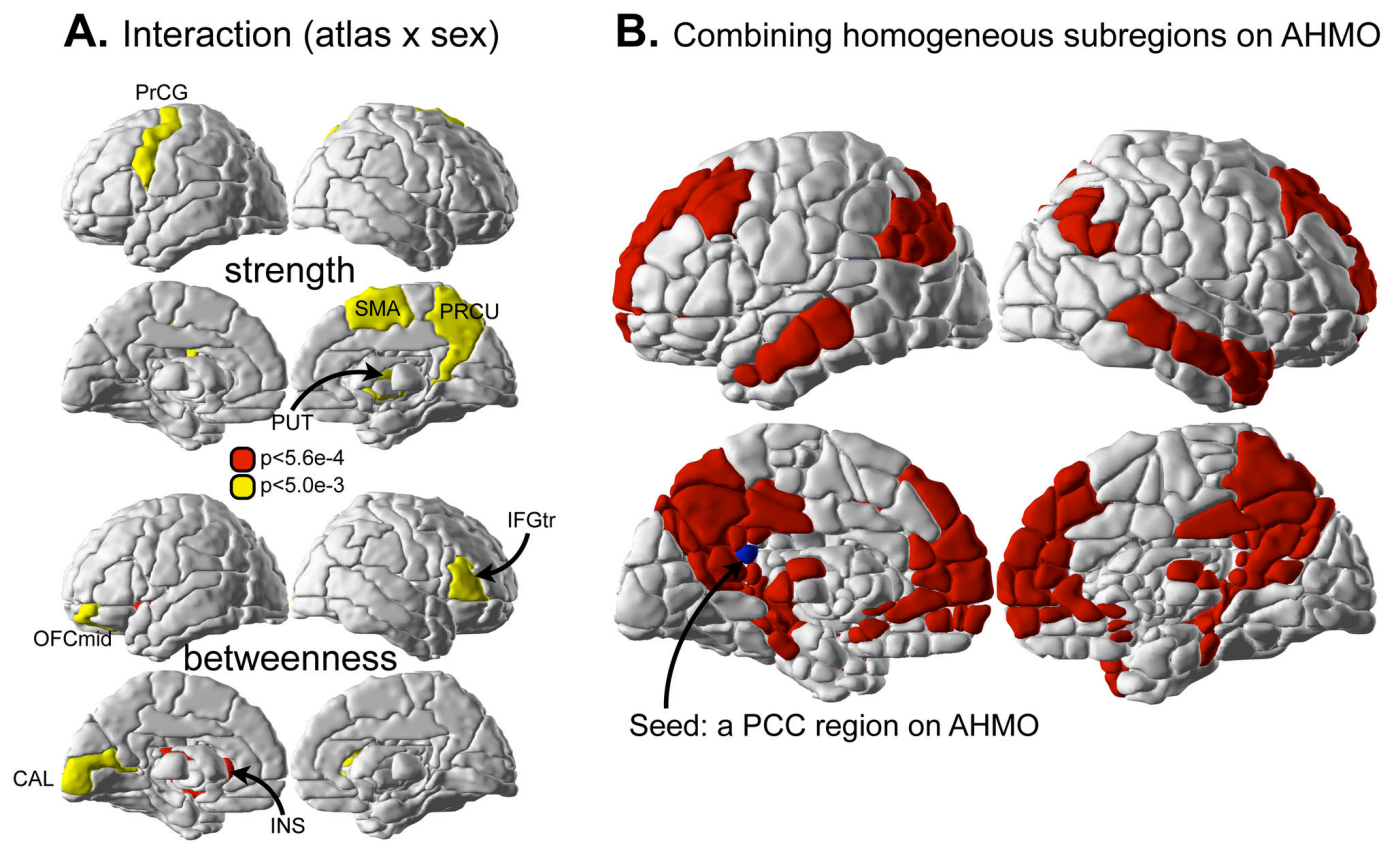
Sex-atlas interactions and functional connectivity maps from the posterior cingulate subregion. (A) Although we did not find any interaction effect in functional connectivity and in global properties (P>0.005), we found a tendency towards interaction between sex and atlas in nodal properties in some brain regions (P<0.005, uncorrected) and significant interactions at the left insula (p<0.05, Bonferroni corrected for all comparisons) in betweenness centrality. They include the left precentral gyrus (PrCG), right supplementary motor area (SMA), right precuneus (PRCU), and right putamen (PUT) in nodal strength and the left middle orbitofrontal cortex (OFCmid), the triangular part of right inferior frontal gyrus (IFGtr), left insula (INS), and left calcarine sulcus (CAL) in betweenness centrality. (B) Subregions that are temporally correlated with a subregion within the posterior cingulate cortex in the AHMO.

We did not find significant interaction effects between sex and atlas in the functional connectivity and global properties according to a criterion of Bonferroni corrected p<0.05. However, we found a significant interaction with betweenness centrality only in the left insula (p<0.05, Bonferroni corrected for all comparisons) (Figure 6A). Tendency towards interaction for nodal strength was shown in the left precentral gyrus, right supplementary motor area, right precuneus, and right putamen, and that for betweenness centrality was shown in the left middle orbitofrontal cortex, the triangular part of right inferior frontal gyrus, left insula, and left calcarine.

### Results according to network sizes, definition of representative time series, and network-constructing thresholds

**Figure 7:**
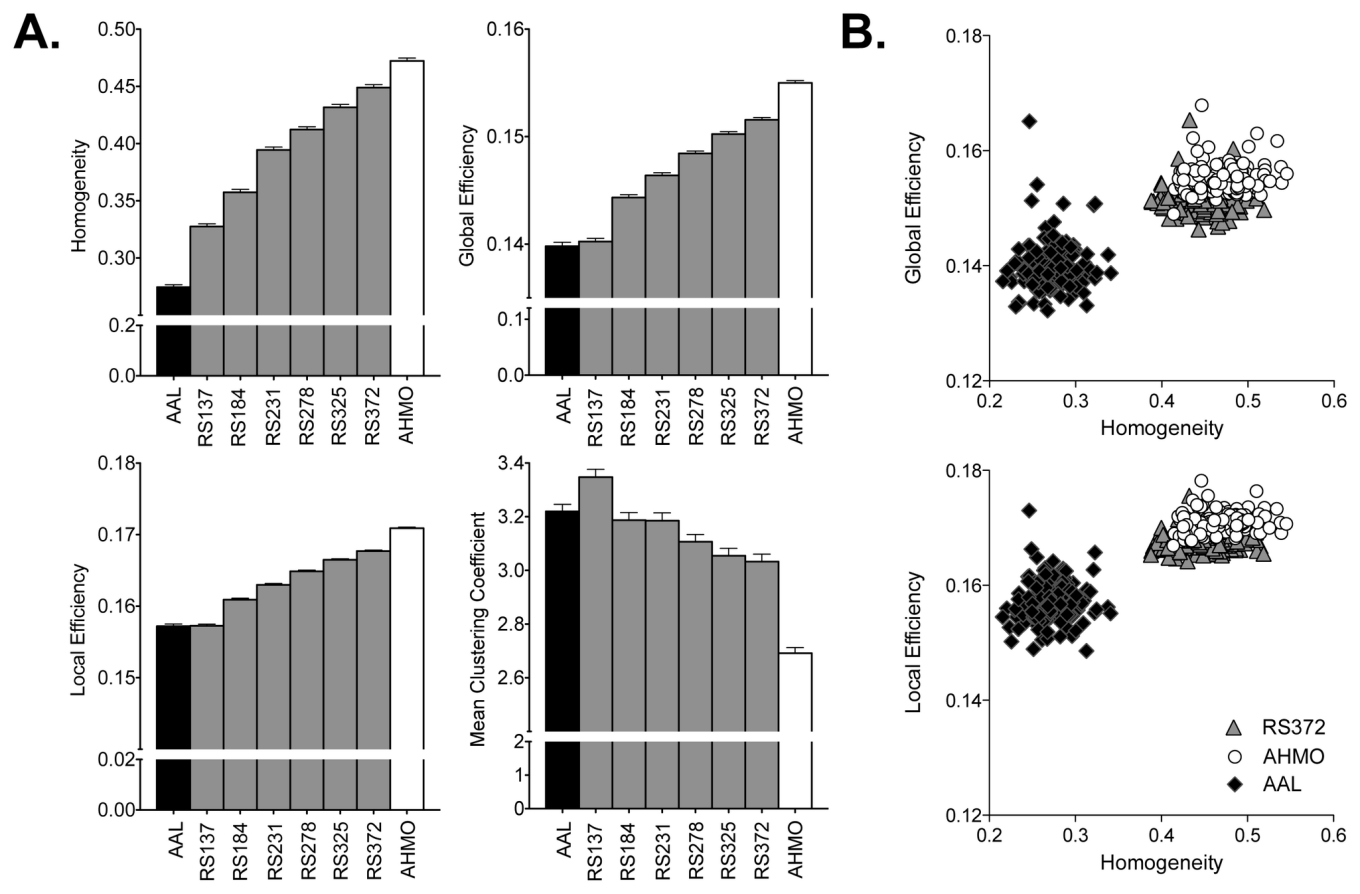
Homogeneity difference and its effect on global properties. (A) Comparison of three atlases (AAL, AHMO and random seeding methods (RS) with different node sizes (RS137, RS184, RS231, RS231, RS372) in terms of mean within-cluster homogeneity and global and local efficiencies and mean clustering coefficients (CC). (B) Results of individual homogeneity and global and local efficiencies for the AAL, RS, and AHMO.When we compared the methods for representing regional time series, we found very high correlation between using the first eigenvariate and the regionally averaged time series (mean correlation coefficient between two time series for all ROIs were distributed from 0.76 (p = 7e-75) to 0.94 (p = 8e-100)). Therefore, we conducted all the comparison in the current study using regionally averaged time series.

In the random seeding atlas, within-cluster homogeneity was higher with increasing number of nodes and they were higher than the homogeneity in the AAL atlas (p<0.05, Bonferroni corrected for all comparisons). In general, increased networks size and thus higher homogeneity showed high global and local efficiencies and low clustering coefficients (except for the random seeding atlas 137). Higher within-cluster homogeneity was found in the AHMO atlas compared to the same-sized random seeding atlas (p=1.6e-106, RS372) (Figure 7A). Accordingly, the AHMO atlas showed significantly higher local (p=1.1e-55) and global efficiencies (p=3.6e-50) than the random seeding atlas (RS372) (Figure 7A-B).

At network-forming thresholds Z=1, 2, and 3, we found a robust linearity results between the AAL-based atlas and AHMO-based atlas in both global and local graph properties (p<0.05, Bonferroni corrected for all comparisons) (Figure 5). However, at Z=0, the linearity between global and local efficiencies and betweenness centrality of the two atlases disappeared and the linearity of nodal strength became distorted (Figure 5).

Figure 6B shows an example of using AHMO to find general functional connectivity architectures as in ICA or voxel-based correlation analysis. Using a seed region within the posterior cingulate cortex, we found that a combination of AHMO subregions, which are temporally correlated but distributed at different AAL ROIs, showed similar network pattern with the default mode network in previous studies conducted at the voxel level (Figure 6B) [25,31,32].

## Discussion

Although graph-theoretical analysis has been widely used to characterize the complex network of the brain and to elucidate its topological organization [5,33], there still exists no consensus for functional node definition. In previous studies using fMRI, many researchers have defined nodes based on anatomical parcellation (e.g., AAL, EZ template in SPM, AFNI template, or Harvard-Oxford template) [14,21,34,35]. However, this study showed that the assumption of homogeneity within anatomically defined nodes is questionable and there exist effects of this inhomogeneity on the network analysis. There are various connectivity-based methods for parcellation of the whole brain such as random seeding using HARDI-based and DTI-based anatomical connectivity [28], and normalized cut [29] and ICA [36] for functional connectivity. However, since these methods are not standardized in the correlation analysis with behavior data using a single atlas same anatomical space, we used AHMO to evaluate anatomical inhomogeneity.

### Functional inhomogeneity and its effect on functional connectivity in the anatomical atlas

In the current study, we found that regions in an anatomical atlas are functionally inhomogeneous and that utilization of such inhomogeneous regions as nodes leads to changes in the functional connectivity and global network properties (Figure 1 and Figure 3).

The functional inhomogeneity shown in Figure 7A-B may be explained by anatomy-function mismatch. The AAL map was constructed based on the sulcus and gyrus and their relative locations. However, it does not mean that these partitions are functionally homogenous within the partition. There are many evidences that have shown inhomogeneous functions within anatomically defined brain regions such as the supplementary motor area [37], thalamus/basal ganglia [38], insula [39], amygdala [40], and orbitofrontal cortex [41]. These evidences have shown that these anatomically defined regions may actually be subdivided into multiple functional subunits through various functional connectivity-based parcellation methods. Current study addressed the issue of inhomogeneity at the whole brain level.

It is important to note that the regional inhomogeneity can result in unexpected regional mean activity, and may conceal region-to-region functional connectivity or create a false-alarm functional connectivity (Figure 3). For example, we found higher functional connectivities in the AAL compared to the AHMO among regions mainly located within the default mode network, sensory-motor network, occipital network, and temporal network. Recent studies [8,42] suggest that although functional connectivity patterns within these networks can be used to separate diverse cognitive states or disease groups, functional connectivity studies using functional inhomogeneous nodes can lead to inaccurate or biased results.

Meanwhile, increased functional connectivities in the AAL may be due to compulsory averaging of signals within a broad brain region, which can lead to larger functional connectivity, especially on the medial side where counterpart regions exist in the other hemisphere. In fact, overestimation of functional connectivities were mainly located on the medial side and existed as bilateral connections.

### Effects of inhomogeneity on graph-theoretical measures

Graph-theoretical analysis has identified key characteristics of functional brain architecture such as the small-worldness and scale-freeness [43,44]. However, most of previous studies have used nodes defined by anatomical parcellation such as the AAL, FreeSurfer map, or Harvard-Oxford atlas [13,14,21], which are likely to be affected by functional inhomogeneity within nodes.

By comparing nodal properties such as nodal strength and betweenness centrality, we observed that using the anatomical parcellation for defining nodes could result in spurious results for regions often referred to as cortical hubs. For example, we found that use of the AAL led to overestimated strength in the posterior cingulate cortex and thalamus/basal ganglia and decreased strength in the sensory-motor regions and the frontal/temporal lobes. Similarly for betweenness centrality, we found underestimated values in the posterior cingulate cortex and decreased values in the thalamus/basal ganglia for the AAL compared to the AHMO. Nodal properties may be highly influenced due to inaccurate inter-regional functional connectivity, since the measures are directly defined by the existence or absence of functional connectivity and its weight.

This study suggests that graph-theoretical analysis driven by functionally inhomogeneous atlas influence key network measures such as global and local efficiency (as measures of functional integration) and nodal strength and betweenness centrality (as measures of functional centrality). Node definition by the anatomical atlas AAL resulted in lower efficiency than in the functional atlas AHMO as the AAL underestimates the efficiency in node-to-whole brain communication. However, the mechanism through which inhomogeneity leads to biased estimations of global and local efficiencies may not be straightforward to resolve.

We acknowledge that the biases in the global and local efficiencies might not be simply attributed to the homogeneity differences. Indeed, we cannot easily differentiate the size effects and homogeneity effects as they are highly correlated. As the network size increases, the mean cluster size decreases and the homogeneity within the cluster increases (Figure 7A). Nevertheless, we found that more homogeneous atlas despite the same network size have generally higher local efficiency but lower mean clustering coefficients. It is obvious that finer parcellation, when it has sufficiently high number of nodes, produces more homogeneous clusters. This was revealed in a previous study using a spectral clustering method, but it did not evaluate the effects of homogeneity on the graph properties [29]. We confirmed the phenomenon in our study, as differences in global properties were smaller between the AHMO and random seeding atlas than between the AHMO and AAL.

### Effect of difference thresholds on linearity and defining representative regional time series

During construction of a brain graph, weak connections are generally eliminated since they may be present by chance [16,45,46], although there exist studies that applied no threshold to build a fully weighted graph [47,48].

In current study, we adopted the general thresholding approach and reported results based on weighted brain graphs thresholded by a critical value of Z=3. However, choice of such threshold value is arbitrary and selecting different thresholds value could cause changes in small-world properties (due to changes in network topology) in weighted graph as well as binary graph [16]. Thus, we evaluated whether or not selection of different thresholds had an effect on the linearity results, by comparing the linearity of network properties in the AAL and AHMO for different network-forming thresholds of Z=0, 1, 2, and 3.

According to our results, linear relationship in both global and local measures was observed for all threshold levels (Z=1, 2, and 3). However, the relationship disappeared when there was no threshold (Z=0). Our results suggest that it is necessary to threshold a weighted graph even if the threshold is low (e.g., Z=1, even though it translates to P>0.05). Given null hypothesis of Z=0, most functional connectivities within a graph might be frequently distributed around Z=0. Such correlations may be regarded as noise that should be eliminated. Therefore, we conclude that there exists a high linearity between AAL and AHMO if their graphs are appropriately thresholded.

Finally, we found very high correlations between the first eigenvariate and regionally averaged time series. Therefore, we conducted all analyses using regionally averaged time series.

### Atlas dependent bias and inter-atlas linearity in graph theoretical measures

In this study, we have demonstrated that using functionally inhomogeneous atlas leads to biased estimation of graph-theoretical measures. Nonetheless, we found high inter-atlas linear relationship or linearity in global graph measures (Figure 5). As global properties are related to scaling of the number of nodes, different node definitions in Achard et al. [16] using small network size (90 nodes) and Van den Heuvel et al. [44] using large network size (10,000 nodes) resulted in only a change in magnitude of small-worldness, not the loss of the general small-world characteristics. Their results may indicate a significant inter-atlas linearity in small-worldness as a global measure, which is similar to significant linearity in global and local efficiency in our study. Therefore, the use of AAL is generally acceptable as long as it is used systematically in evaluating the global network properties using an atlas (e.g., group comparison or correlation analysis with behavior data using an anatomical atlas).

We also found such linearity in nodal strength but linearity between betweenness centralities of the AAL and AHMO atlases was insignificant in some brain regions such as the default mode network, sensory-motor regions, occipital regions, temporal regions, and subcortical regions. Although they have been referred to as cortical hub regions [8,49,50], the regions within the default mode network (e.g., the anterior cingulate cortex, posterior cingulate cortex, and precuneus) should be carefully treated when conducting an analysis of betweenness centrality (Figure 5).

Meanwhile, use of functionally inhomogeneous atlas does not significantly affect group-level results as we found no significant interaction effect (atlas x sex) in nodal properties. These results suggest that use of an inhomogeneous atlas may not lead to critical distortion in the results of group-level comparison of graph-theoretical analysis.

Therefore, we conclude that use of anatomical atlas, despite its inhomogeneity, is generally acceptable except for atlas-dependent betweenness centrality, if we use it for a group comparison study or a correlation analysis with behavior data when a single atlas is used for all data.

### Use and characteristics of AHMO: goal, novelty, advantage, and limitation

In parallel with utilizing anatomical atlas, recent functional connectome studies have used diverse functional atlases [51-55] derived from many different methods; for example, meta-analysis results from task-based functional studies [51,53], high-dimensional ICA [55], clustering of time series [52], or clustering of graphs derived from time series [54]. All these methods are independent of anatomy, which makes it difficult to directly compare the performance of the anatomical atlas in the application of functional networks as conducted in the current study.

In this study, we proposed AHMO to generate an anatomically-constrained functionally homogeneous atlas and to investigate the potential problems in use of anatomical atlas. The method uses modularity optimization to subdivide the whole brain into functionally homogenous subunits.

Modularity optimization, which originates from graph theoretical analysis [26], has been mainly used to discover functional subunits called “modules” or to investigate their hierarchical relationship in the whole brain as a fully data-driven approach [56-58]. Modularity optimization is particularly appealing because it does not require a pre-defined number of clusters as an input, compared to other clustering methods such as ICA [59], normalized cut [29], and k-means [52].

AHMO applies modularity optimization to voxel-level fMRI time series on anatomy-constrained ROIs (regions in the AAL), which is different from previous studies using modularity optimization without any atlas or seed brain regions [36]. AHMO using hierarchical subdivision is more precise than global modularity optimization method (cf. Valencia et al. [36]).

Since AHMO may be considered to be hierarchically nested within an anatomical map, an atlas that results from applying this method can be easily interpreted. As described above, AHMO differs from various functional parcellation methods such as ICA [59], normalized cut [29], and k-means [52], all of which partition the whole brain or its parts based purely on functional information. With anatomical constraint, AHMO allows easy comparison of functional and anatomical partitioning. Also, it is convenient to understand subregions based on an anatomical atlas because most previous neuroimaging studies are based on anatomical atlases. If one needs to derive functionally homogeneous clusters as in many previous studies [25,31,32], it is possible to combine subregions distributed at different AAL ROIs according to its temporal dynamic to construct functionally homogeneous maps (Figure 6B). For example, Figure 6B shows all combined subregions that are temporally correlated with a region within the posterior cingulate cortex in the AHMO map. Resulting map has a similar pattern to the default mode network in previous studies conducted at the voxel level [25,31,32]. The anatomical constraining strategy of AHMO is similar to that of a recent paper independently conducted by Blumensath et al. [60], who applied normalized cut algorithm. However, the authors did not evaluate the effect of homogeneity on the graph properties as was done in this study.

In generating the AHMO map, we used spatially smoothed functional data to minimize the misalignment errors across subjects as is often the case in the group-level voxel-wise parcellation [29,37,38,53,61]. A recent paper on the whole brain functional parcellation [29] addressed this issue and showed that data smoothed with 6-mm full-width half-maximum kernel were not over-smoothed.

A preliminary analysis of unsmoothed data with motion derivatives as additional covariates showed no significant difference from the current results with smoothed data in the respect of the effects of inhomogeneity on the network properties and their linearity between AAL and AHMO methods (Figure S1 and Figure S2).

In addition to the smoothing effect, the head motion effect is often an issue in the functional network analysis [62-64]. Since the initial focus of the current study was to show the effects of within-node inhomogeneity on the graph properties using conventional procedures, we regressed out the head motion effects using six rigid motion parameters [29,54,60,61,65]. As shown in the supplementary analysis with motion derivatives as covariates [62] (in Figure S1 and Figure S2), the effects of motion derivatives might not significantly affect the current results.

Among many available anatomical atlases, we evaluated only the AAL atlas. We conjectured that the current results would be similar across different anatomical atlases, as the anatomical atlases are based on sulcus-gyrus landmarks and may not be significantly different among them. As the evaluation of the inhomogeneity effect on the graph properties was the main focus of the current study, we did not directly compare the performance of the AHMO with other parcellation methods, which will be the subject of a future study.

## Conclusion

Current study revealed that an anatomical parcellation such as AAL has functional inhomogeneity within its regions and may lead to biased estimation of functional connectivity and graph-theoretical analysis. This study also revealed high linearity between graph analysis results based on anatomical and functional atlases. Therefore, we concluded that use of anatomical atlas, despite its inhomogeneity, is generally acceptable for group comparison studies and correlation analysis with behavior data using a single atlas, although some nodal properties such as betweenness centrality should be analyzed with caution. To address these problems, we proposed an anatomy-constrained hierarchical modularity optimization algorithm, which generates a functionally homogeneous atlas.

## Supporting Information

Figure S1 **Difference in functional homogeneity and global network properties analyzed from unsmoothed data with motion derivative information as covariates.** The results are similar to results with smoothed data presented in Figure 7. Comparison of three atlases (AAL, new AHMO (number of nodes=358) and random seeding methods (RS) with different node sizes (RS150, RS200, RS250, RS300, RS358) in terms of mean within-cluster homogeneity, global and local efficiencies and mean clustering coefficients.
(TIFF)

Figure S2 **Linearity between AAL and AHMO according to network-forming thresholds from unsmoothed data with motion derivative information as covariates.** At different network-forming thresholds of Z=1, 2, and 3, the AAL-based and AHMO-based approaches showed high linearity in global network properties. These results are similar to the results analyzed using smoothed data in Figure 5.
(TIFF)
