# Correction: Evaluation of Node-Inhomogeneity Effects on the Functional Brain Network Properties Using an Anatomy-Constrained Hierarchical Brain Parcellation

**DOI:** 10.1371/annotation/041d3c2c-5071-4abb-8727-67e4948a1ce2

**Published:** 2013-10-24

**Authors:** Bumhee Park, Jeong Hoon Ko, Jong Doo Lee, Hae-Jeong Park

There was an error in the Funding statement. The correct version of the Funding statement is available below.

This research was supported by the Original Technology Research Program for Brain Science through the National Research Foundation of Korea (NRF) funded by the Ministry of Education, Science and Technology (20100018839 for HJP). The funders had no role in study design, data collection and analysis, decision to publish, or preparation of the manuscript. 

