# Supplementary figures and images for: Evaluation of Node-Inhomogeneity Effects on the Functional Brain Network Properties Using an Anatomy-Constrained Hierarchical Brain Parcellation
Source: PLoS One. 2013 Sep 18;8(9):e74935. doi: 10.1371/journal.pone.0074935 (PMC3776746; doi:10.1371/journal.pone.0074935)

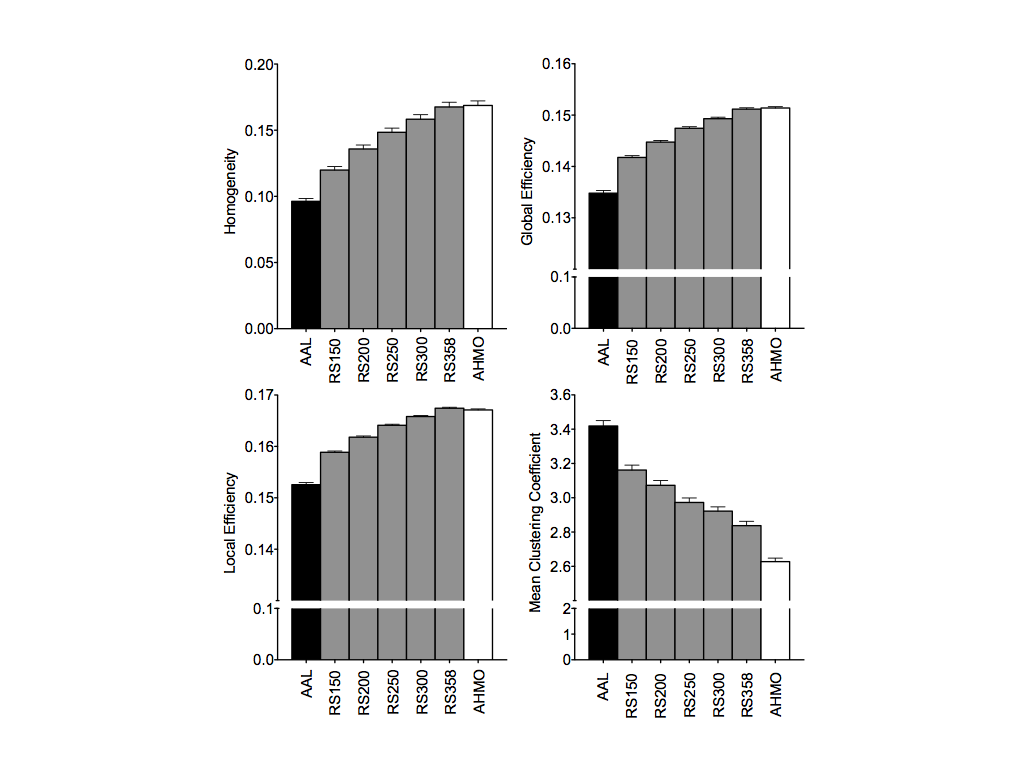

Supplement: Figure S1 — Difference in functional homogeneity and global network properties analyzed from unsmoothed data with motion derivative information as covariates. The results are similar to results with smoothed data presented in Figure 7. Comparison of three atlases (AAL, new AHMO (number of nodes=358) and random seeding methods (RS) with different node sizes (RS150, RS200, RS250, RS300, RS358) in terms of mean within-cluster homogeneity, global and local efficiencies and mean clustering coefficients. (TIFF) [file pone.0074935.s001.tiff]

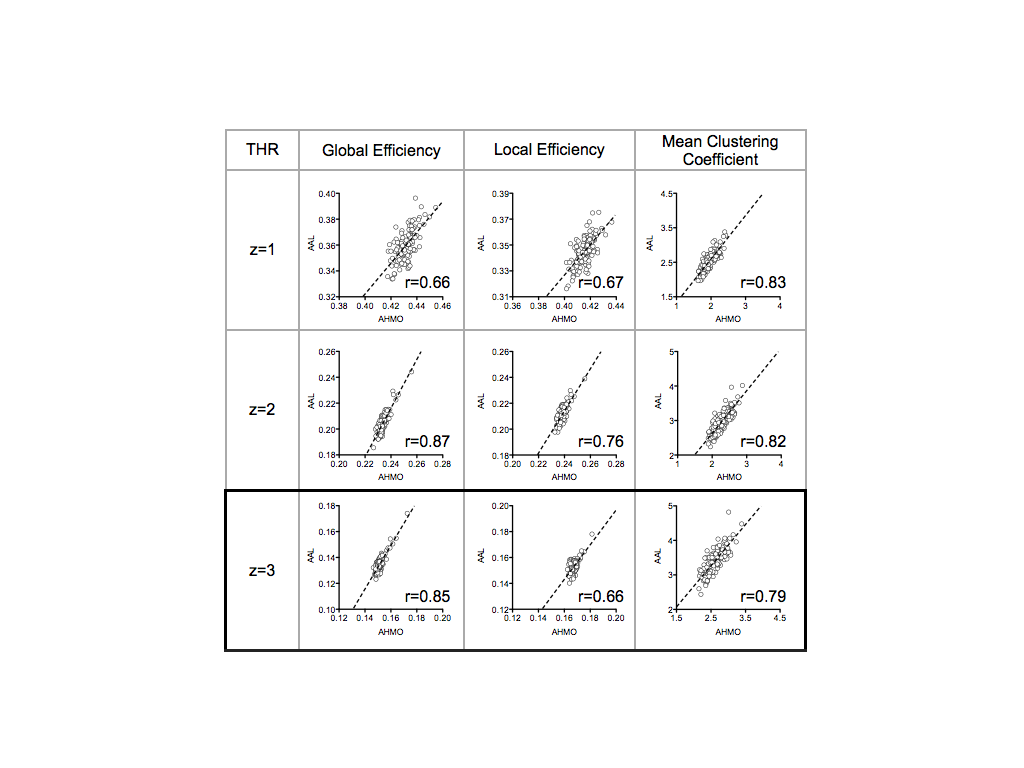

Supplement: Figure S2 — Linearity between AAL and AHMO according to network-forming thresholds from unsmoothed data with motion derivative information as covariates. At different network-forming thresholds of Z=1, 2, and 3, the AAL-based and AHMO-based approaches showed high linearity in global network properties. These results are similar to the results analyzed using smoothed data in Figure 5. (TIFF) [file pone.0074935.s002.tiff]
